# *Annona Muricata L.* extract restores renal function, oxidative stress, immunohistochemical structure, and gene expression of TNF-α, IL-β1, and CYP2E1 in the kidney of DMBA-intoxicated rats

**DOI:** 10.3389/fphar.2024.1348145

**Published:** 2024-02-01

**Authors:** Mohamed M. Zeweil, Asmaa F. Khafaga, Sahar F. Mahmoud, Lamiaa Wasef, Hamida Saleh, Attaa. M. Abd Elrehim, Naglaa F. Bassuoni, Maha Abdullah Alwaili, Nizar H. Saeedi, Hanan A. Ghoneim

**Affiliations:** ^1^ Department of Biochemistry, Faculty of Veterinary Medicine, Damanhour University, Damanhour, Egypt; ^2^ Department of Pathology, Faculty of Veterinary Medicine, Alexandria University, Alexandria, Egypt; ^3^ Department of Cytology and Histology, Faculty of Veterinary Medicine, Damanhour University, Damanhour, Egypt; ^4^ Department of Pharmacology and Therapeutics, Faculty of Veterinary Medicine, Damanhour University, Damanhour, Egypt; ^5^ Department of Toxicology and Forensic Medicine, Faculty of Veterinary Medicine, Damanhour University, Damanhour, Egypt; ^6^ Department of Physiology, Faculty of Veterinary Medicine, Damanhour University, Damanhour, Egypt; ^7^ Department of Anatomy and Embryology, Faculty of Veterinary Medicine, Alexandria University, Alexandria, Egypt; ^8^ Department of Biology, College of Science, Princess Nourah bint Abdulrahman University, Riyadh, Saudi Arabia; ^9^ Department of Medical Laboratory Technology, Faculty of Applied Medical Sciences, University of Tabuk, Tabuk, Saudi Arabia

**Keywords:** graviola, nephrotoxicity, MDA, DMBA, TNF-α, IL-1β, CYP2E1, antioxidant enzymes

## Abstract

**Introduction:** 7,12-dimethylbenz (a) anthracene (DMBA) is a harmful polycyclic aromatic hydrocarbon derivative known for its cytotoxic, carcinogenic, and mutagenic effects in mammals and other species. Annona muricata, L. (Graviola; GRV) is a tropical fruit tree traditionally well-documented for its various medicinal benefits. This investigation is the first report on the potential antioxidant and antinfammatory reno-protective impact of GRV against DMBA-induced nephrotoxicity in rats.

**Methods:** Forty male albino rats were allocated into four equal groups (n = 10). The 1st group served as the control, the 2nd group (GRV) was gastro-gavaged with GRV (200 mg/kg b.wt), the 3rd group (DMBA) was treated with a single dose of DMBA (15 mg/kg body weight), and the 4th group (DMBA + GRV) was gastro-gavaged with a single dose of DMBA, followed by GRV (200 mg/kg b.wt). The GRV administration was continued for 8 weeks.

**Results and Discussion:** Results revealed a significant improvement in renal function, represented by a decrease in urea, creatinine, and uric acid (UA) in the DMBA + GRV group. The antioxidant potential of GRV was confirmed in the DMBA + GRV group by a significant decline in malondialdehyde (MDA) and a significant increase in catalase (CAT), superoxide dismutase (SOD), glutathione S transferase (GST), and reduced glutathione (GSH) compared to DMBA-intoxicated rats; however, it was not identical to the control. Additionally, the antiinflammatory role of GRV was suggested by a significant decline in mRNA expression of cytochrome P450, family 2, subfamily e, polypeptide 1 (CYP2E1), tumor necrosis factor-alpha (TNF-α), and interleukin 1 beta (IL-1β) in the DMBA + GRV group. Moreover, GRV improved the histopathologic and immunohistochemical expression of TNF-α, CYP450, and IL1β in DMBA-intoxicated kidney tissue. Conclusively, GRV is a natural medicinal product that can alleviate the renal injury resulting from environmental exposure to DMBA. The reno-protective effects of GRV may involve its anti-inflammatory and/or antioxidant properties, which are based on the presence of phytochemical compounds such as acetogenins, alkaloids, and flavonoids.

## 1 Introduction

Although modern industrial development has significantly improved human life, it has also raised various environmental concerns. Industrial pollutants pose a concern for health since they are present in the soil, water, air, and food (Chen et al., 2022). Incomplete combustion of fossil fuels in internal combustion engines, coke manufacturing, household heating, and incineration, as well as natural occurrences like forest fires and volcanoes, all result in the creation of polycyclic aromatic hydrocarbons (PAHs) (Chen et al., 2022)**.** PAHs are a group of organic pollutants released into the environment in large quantities, mostly due to human activities (Barrett et al., 2022). 7,12-dimethylbenz (a) anthracene (DMBA) is a harmful PAH derivative that has well-documented cytotoxic, carcinogenic, mutagenic, and immunosuppressive effects on mammals and other animals (Fidianingsih et al., 2022). In previous studies, DMBA led to the potential growth of malignant tumors in the bladder, kidney, liver, and brain (John et al., 2022)**.** Moreover, it has altered liver metabolic enzymes and induced harmful renal disorders (Molnar et al., 2022)**.** The adverse initiator and promoter properties of DMBA are attributed to its produced metabolites, which can alkylate DNA or other cellular macromolecules (Mathiyazhagan et al., 2022), trigger the formation of reactive oxygen species (ROS) (Hosny et al., 2021)**,** alter behavior and cellular dysfunction (Li et al., 2016), and initiate carcinogenicity, particularly in animal research and cancer models (Zhang et al., 2022).

Generally, environmental pollutants raise the risk of renal, cardiac, and pulmonary disorders (Lu et al., 2022). Various environmental xenobiotic toxicants attack the kidney, resulting in nephrotoxicity (Ren et al., 2022). Currently, nephrotoxicity and chronic renal disease have been recognized as public health problems (Sanchez et al., 2022). The development of glomerular and tubular epithelial cell damage could be attributed to the disruption of cell membrane integrity and normal cellular processes in mitochondria (Dakrory et al., 2015b). DMBA has been reported to induce severe nephrotoxicity, which is characterized by renal tubular necrosis, nuclear chromatin condensation, injured mitochondria, and increased lysosome number (Ozturk et al., 2006).

Traditionally, several disorders have been treated with natural medicines. Therefore, it is important to find natural substances with potent renopreventive properties with minimum or no side effects. The *Annona muricata L.* fruit tree, often known as the soursop or graviola (GRV), is mainly grown in tropical regions of Central and South America (Moghadamtousi et al., 2014). GRV is a natural antioxidant supplement that improves body performance under stressful conditions (Samarghandian et al., 2013). In the tropics, this plant’s bark, fruit seeds, leaves, and root are all used in herbal medicine (Sabra and Ahmed, 2018). According to available research, *A. muricata* extract helps prevent kidney damage because it contains tannins, glycosides, flavonoids, and saponins. These compounds are used to treat a variety of illnesses, including renal impairment (Arthur et al., 2011; Hassan et al., 2019). The graviola extract’s phytochemical analysis revealed many valuable secondary metabolites, such as tannins and steroids (El-Shahat et al., 2021). In numerous studies, GRV has been shown to protect against oxidative stress, enhance antioxidant enzymes such as catalase, superoxide dismutase, glutathione peroxidase, and glutathione, improve blood lipid levels, decrease LDL, total cholesterol, and triglycerides, increase HDL, and exert a wide range of beneficial effects, including anticonvulsive, anticancer, anti-arthritic, anti-malarial, hepatoprotective, and antidiabetic ones (Rady et al., 2018; Shukry et al., 2020; El-Shahat et al., 2021). Recent researches support the usefulness of GRV in the prevention of cancer and degenerative diseases, including renal disease, due to the presence of potent antioxidants such as polyphenols and flavonoids, which provide defense against the accumulation of reactive oxygen species (ROS) (Alam et al., 2014). These ROS are key signaling molecules that play a crucial role in the development of pro-inflammatory disorders (Mittal et al., 2014). Findings from recent studies confirmed that the presence of anti-inflammatory agents in GRV extracts, such as alkaloids, saponins, flavonoids, and tannins, which inhibit COX-2 and prostaglandin synthesis, can normalize the level of proinflammatory mediators (Serafini et al., 2010; Foong and Hamid, 2012; Laksmitawati et al., 2016; Shukry et al., 2020).

To date, few studies have investigated the anti-inflammatory effect of GRV extract against inflammatory renal damage. No studies are available concerning its impact on the cytochrome P450 family 2 subfamily E member 1 (CYP2E1). Thus, the current study aims to evaluate the alterations in renal function, oxidative/antioxidative status, gene expression of NF-α, CYP2E1, and IL-β1, as well as immunohistochemical expression of TNF-α, IL1β, and CYP450 in renal tissues of DMBA-intoxicated rats, and to assess, for the first time, the possible therapeutic impacts of GRV in DMBA-induced nephrotoxicity.

## 2 Materials and methods

### 2.1 Chemicals

DMBA was supplied by Sigma Chemical Co. (St. Louis, MO, United States). Forward and reverse primers for cytochrome P450, family 2, subfamily e, polypeptide 1 (CYP2E1), tumor necrosis factor alpha (TNF-α; 24835), interleukin 1 beta (IL-1β; I2393), and β-actin were purchased from Sigma Chemical Co. (St. Louis, MO, United States). Graviola (GRV) capsules were purchased from Inkanatural (Lima, Peru) (Tables 1, 2). Kits for catalase (CAT) (Cat. No. CAT 25 17), superoxide dismutase (SOD) (Cat. No. SD 25 21), glutathione-S-transferase (GST) (Cat. No. GT 2519), reduced glutathione (GSH) (Cat. No. RG 2523), and malondialdehyde (MDA) (Cat. No. MD 2529) were purchased from the Bio-Diagnostics Co. (Cairo, Egypt). Biochemical test kits for urea (10505), creatinine (creat; 10053), and uric acid (UA; 10694) were provided by Human Diagnostic Worldwide Co. (Wiesbaden, Germany).

**TABLE 1:** The qualitative analysis of an aqueous extract of graviola

Phytochemical components	Aqueous extract of graviola
Acetogenins	++
Alkaloids	++
Flavonoids	++
Terpenoids	+
Saponins	-
Tannins	+
Phenols	+
Reducing sugar	+

**TABLE 2:** Mean values (%) of phytochemical screening of graviola showing mean ± SEM.

Phytochemical components	% Composition
Acetogenins	25.72 ± 0.018
Alkaloids	15.35 ± 0.016
Flavonoids	13.14 ± 0.013
Terpenoids (Sesquiterpene)	0.61 ± 0.006
Phenols	1.32 ± 0.009
Tannins	0.96 ± 0.007
Reducing sugar	0.21 ± 0.003

### 2.2 Characterization of phenolic compounds: HPLC analysis

The phenolic reference standard for HPLC was used to identify phenolic components in the methanolic and aqueous extract of A. muricata (Sakakibara et al., 2003). The following gradient elution program was used, with solution A (50 mM sodium phosphate in 10% methanol; pH 3.3) and solution B (70% methanol) being eluted at 35°C: A solution’s percentages are as follows: 0–15 min–100%; 15–45 min–70%; 45–65 min–65%; 65–70 min–60%; 70–95 min–50% of Solution A; 95–100 min–0% of Solution A. 20 μL was the injection volume, and the flow rate was 1 mL min^−1^. Table 3 shows the different wavelengths (around λ max) at which detection were tested for different phenolic compounds.

**TABLE 3:** Major phenolic compounds identified in methanol and aqueous extracts of A. Muricata by HPLC.

*A. muricata* aqueous extracts	λa (nm)	*A. Muricata* methanolic extract	λa (nm)
Tangeretin	320	Luteolin	320
**rolliniastatin-1**,2	220		
**Annonacin**	220		
Homoorientin	320	Homoorientin	320
Luteolin	320	Quercetin	370
Genistein	250	Daidzein	250
Glycitein	250	Coumarid acid	320
Catechin	280	Isoferulic acid	320
Emodin	250		

### 2.3 Animals and ethical statement

Forty male Wistar albino rats (average weight 130 ± 10 gm, average age of 6 weeks old) were obtained from the Animal House Colony, National Research Centre, Cairo, Egypt, were kept for 2 weeks for adaptation. They kept under a 12-h light-and-darkness cycle and controlled temperature of 21°C ± 2°C. Throughout the experiment, animals received standard rodent diets (23% crude protein, 7% crude fiber, 3.0% crude fat, 8% acid insoluble ash, 1%–2.5% calcium, 0.5%–1% phosphorus, and 0.9% sodium) and free access to water. The animal studies were ethically approved (DMU/VetMed-2023/020) by the Hygiene and Preventive Medicine research committee, Faculty of Veterinary Medicine, Damanhur University, Egypt.

### 2.4 Experimental design

After acclimation, rats were randomly assigned to four groups (10 rats each). The 1st group was served as a control and administered a single dose of sesame oil (1 mL; as a vehicle for DMBA), followed by a daily dose of distilled water (1 mL; as a solvent for GRV), which started on the 2nd day and continued for 8 weeks via gastric gavage. The 2nd group (GRV) was gastro-gavage with a single dose of sesame oil (1 mL), followed by GRV (200 mg/kg b.wt.) according to Florence et al. (2014) from the second day, and continued for 8 weeks. The 3rd group (DMBA) was treated with a single dose of DMBA (15 mg/kg body weight) dissolved in 1 mL of sesame oil, according to Dakrory et al. (2015a). Rats in the 4th group (DMBA + GRV) were gastro-gavaged with a single dose of DMBA (15 mg/kg body weight), followed by GRV (200 mg/kg body weight), which started from the second and continued for 8 weeks.

At the end of the experiment, rats were anesthetized given 87 mg ketamine/kg of body weight and 13 mg xylazine/kg, beginning 10 to 15 min after simultaneous injection and lasting 15 to 30 min. Rats also were sacrificed by cervical dislocation (Kumar and Clover, 2015). The blood samples were collected by heart puncture before the abdominal incision. Serum samples were separated by centrifugation at 1008 G-force and stored at −20°C for further biochemical analysis of renal function markers. Following euthanasia, the kidney was immediately collected, weighed, and divided into three parts in a saline phosphate buffer (PBS) solution. The first part was kept at −80 for gene expression studies. The second part was kept in a buffered formalin solution (10%) for histopathological examination and immunohistochemical analysis, while the third part was homogenized and kept at −20°C for further evaluation of malondialdehyde (MDA) and antioxidant parameters.

### 2.5 Assessment of renal function in serum

Markers for renal functions (urea, creatinine, and uric acid) were spectrophotometrically assessed using commercially available diagnostic kits (Human Scient Co., Germany), according to Chescheir, (2019), Chromy et al. (2008), and Kessler and Siekmann, (1999).

### 2.6 Assessment of oxidative/antioxidative markers in homogenized renal tissues

Following euthanasia, renal tissues were immediately collected, weighed, homogenized, and centrifuged at 4,000 rpm for 15 min (137 mM NaCl, 2,7 mM KCl, ten mM Na_2_HPO_4_, two mM KH_2_PO_4_, pH 7,4). The supernatants were used to assess catalase (Aebi, 1984), SOD (Wang et al., 2016), GST (Habig et al., 1974), GSH (Beutler et al., 1963), and MDA (Ohkawa et al., 1979) according to the manufacturer’s instructions using the commercially available kits (Bio-diagnostic Co., Cairo, Egypt).

### 2.7 Assessment of mRNA expression of TNF-α, CYP2E1, and IL-β1

The mRNA has been isolated, according to Boom et al. (1990). Following this, cDNA is synthesized using the RevertAidTM Synthesis Kit, according to Wiame et al. (2000). Gene expression of CYP2E1, TNF-α, and IL1β was performed in the presence of β-actin as a housekeeping gene (Longo et al., 1990) (Table 4). The master mix was prepared, and the reaction was performed in a thermal cycler (MyCycler, Bio-Rad, Germany). The whole volume of the reaction was 25 μL for each gene of interest. The length of the PCR primers was 18-22 bp**.** The target gene’s relative quantification to the reference was determined using the ΔΔCT method (Livak and Schmittgen, 2001).

**TABLE 4:** List of the sequences of the primers.

Gene	Accession code	Forward primer	Reverse primer
Tnf-α	NM_012675.3	CCA​CGT​CGT​AGC​AAA​CCA​C	TGG​GTG​AGG​AGC​ACG​TAG​T
Cyp2e1	NM_031543.2	AAGCGCTTCGGGCCAG	TAG​CCA​TGC​AGG​ACC​ACG​A
IL-1β	NM_031512.2	ACC​CAA​GCA​CCT​TCT​TTT​CCT​T	TGC​AGC​TGT​CTA​ATG​GGA​ACA​T
β-actin	NM_031144.3	TCT​TCC​AGC​CTT​CCT​TCC​TG	CAC​ACA​GAG​TAC​TTG​CGC​TC

### 2.8 Histopathological examination

The rats' kidneys were dissected after an incision in the abdominal wall. The collected specimens were thoroughly washed and fixed in a neutral buffered formalin solution (10%) for 48 h. The fixed specimens were then processed via the conventional paraffin embedding technique. Briefly, samples were washed in distilled water for 30 min, dehydrated in an ascending grade of ethyl alcohol, cleared in several changes of xylene, and then embedded and blocked out in paraffin wax. Four µm thick sections were microtomed and stained with hematoxylin and eosin (H & E), periodic acid Schiff reaction (PAS), and a combination of periodic acid Schiff reaction (PAS) with Alcian Blue (AB) according to Bancroft and Gamble (2008) and Dosumu et al. (2021). The PAS technique was used for the demonstration of glycoproteins and mucins (neutral and acid mucin) and for the visualization of basement membranes. Alcian Blue/PAS is used to differentiate between neutral mucins and acid mucins, where the expression patterns of neutral and acid mucins may indicate certain pathological lesions if present. A blind pathologic examination was performed by two expert pathologists. A digital camera (Leica EC3; Leica, Germany) connected to a light microscope (Leica DM500) captured several representative photomicrographs. The extent of kidney injury in H&E sections was assessed by a semi-quantitative scoring system; ten rats from each group had their kidneys inspected and graded for various pathologic lesions in the glomerulus, renal tubules, and interstitium. Five random fields were evaluated for each rat. The degree of severity of the detected histopathologic lesions was estimated according to Khafaga et al. (2021). Four levels of severity were used in the scoring system: no histologic change (−), mild (+), moderate (+), and severe (+++) pathologic changes. The individual score for each rat was estimated, and then the grading decision was determined by calculating the median score for each group. For the quantitative histomorphometric analysis of PAS and the Alcian Blue/PAS staining technique, original representative micrographs (×100) were captured from five randomly selected fields in each section for the quantitative histomorphometric analysis of staining optical denisty. ImageJ software (v1.46r, NIH, Bethesda, MD, United States) was used to estimate the differences in optical densities, which were represented by the difference in distribution of positively reacted cells. Optical densities were evaluated as the percentage of the mean number of pixels versus the correlated value at which the pixel of the respective intensity was present (Onouchi et al., 2013; Varghese et al., 2014).

### 2.9 Immunohistochemical analysis

Immunohistochemical analysis was performed in accordance with Tawfik et al. (2021). In brief, the prepared sections were dewaxed, rehydrated in a graded series of ethyl alcohol, exposed to antigen retrieval for 20 min via citrate buffer, deactivated for endogenous peroxidase by H2O2 (3%) for 5 min, and blocked for the non-specific reaction by a 60-min incubation with normal goat serum (10%). After that, sections were incubated overnight with anti-TNF-α antibody (Abcam, Cat. Ab220210, Cambridge, United Kingdom), anti-CYP450 antibody (Abcam, Cat. Ab197053, Cambridge, United Kingdom), and anti-IL1β antibody (Abcam, Cat. Ab 9722, Cambridge, United Kingdom) at 4°C. Sections were then incubated with biotin-conjugated goat anti-rabbit IgG antiserum (Histofine kit, Nichirei Corporation, Japan) for 60 min, followed by 30 min of incubation with streptavidin-peroxidase conjugate (Histofine kit, Nichirei Corporation, Japan). The streptavidin-biotin complex was visualized by a 3,3′-diaminobenzidine tetrahydrochloride (DAB)-H2O2 solution. Counterstaining with Mayer’s hematoxylin solution was done. Finally, representative photomicrographs were captured from each group by a digital camera (EC3, Leica, Germany) connected to a light microscope (Leica DM500). Original representative photomicrographs (×**1**00) were taken from five randomly selected fields in each section for the quantitative histomorphometric analysis of immunostaining intensity. ImageJ software (v1.46r, NIH, Bethesda, MD, United States) was used to estimate the differences in optical densities, which were represented by the difference in distribution of positively immunoreacted cells. Optical densities were evaluated as the percentage of the mean number of pixels versus the correlated value at which the pixel of the respective intensity was present (Onouchi et al., 2013; Varghese et al., 2014).

### 2.10 Statistical analysis

The statistical analysis was carried out using SPSS program version 20 (SPSS, Richmond, VA, United States), with the data presented as means ± SE. Data were analyzed using one-way ANOVA, and the significant differences between treatments were assessed by a *post hoc* review of the Duncan test at *p* < 0.01. The assumptions of observation independence as well as the normality and homogeneity of variances were assessed by Shapiro-Wilk test before conducting the ANOVA test.

## 3 Results

During the experiment, rats in the DAMP group showed symptoms of decreased body weight, loss of appetite, and ruffled hair. Only one rat died on the tenth day of the experiment. On the contrary, the control group and other treated groups did not suffer from any apparent symptoms or deaths. The therapeutic effect of GRV on various parameters will be presented below.

### 3.1 Phytochemicals identified and HPLC analysis

The GRV phytochemical screening data, detailing the chemical constituents in plant extracts, are presented in Tables 1, 2. This screening is essential for identifying specific phytochemicals in the extracts, particularly in the methanol and aqueous extracts of *Annona muricata* leaves. These compounds were identified using High-Performance Liquid Chromatography (HPLC) analysis, a technique that separates, identifies, and quantifies compounds in a mixture. For accurate identification, we referenced a library with analytical properties of over 100 phenolic standards, established by Sakakibara et al. 2003. This library includes crucial data like maximum absorption wavelength (λ max), retention time, and calibration limits. Our HPLC results were then compared to these standard values, ensuring precise compound identification. The identified phenolic compounds in the extracts, along with their corresponding wavelengths in nanometers (nm), are listed in Table 3.

### 3.2 The therapeutic impact of GRV on renal functions of DMBA-intoxicated rats

As shown in Table 5, serum urea and creatinine levels were significantly increased in the DMBA-treated group compared to the control group. At the same time, oral administration of GRV to DMBA-intoxicated rats caused a significant decrease (*p* < 0.0001) in their concentration compared to the corresponding DMBA groups; however, it still significantly increased (*p* < 0.0001) compared to the control group. A similar result was reported for uric acid (*p* < 0.001). There was no discernible difference between the control group and the GRV administration alone.

**TABLE 5:** The therapeutic impact of graviola (GRV) on some renal markers (urea, creatinine, and uric acids) of rats intoxicated with DMBA and treated with graviola compared to control rats.

Group	UREA	CREAT	UA
Control	31.68 ± .958^c^	0.57 ± .09^c^	2.83 ± .072^c^
GRV	31.3400 ± 1.41^c^	0.66 ± .10^c^	2.68 ± .67^c^
DMBA	122.52 ± 1.01^a^	2.43 ± .20^a^	8.27 ± .72^a^
DMBA + GRV	62.1300 ± 1.82^b^	1.64 ± .13^b^	5.44 ± .79^b^
*p*-value	0.0001	0.0001	0.001

Data presented as means ± SEM for ten rats in each group. The significant change was at *p* < 0.05. Means within the same column, which carry different superscript letters (a, b and c)**,** are significantly different (*p* < 0.01). (N = 10). DMBA: 7, 12-Dimethylbenz (a) anthracene; GRV: graviola.

### 3.3 The therapeutic impact of GRV on antioxidant markers in renal tissues of DMBA -intoxicated rats

As shown in Table 6, CAT and SOD enzymatic activities and GSH non-enzymatic levels were significantly decreased (*p* < 0.0001) in the DMBA group as compared to the corresponding control groups. Whereas, oral administration of GRV in DMBA-intoxicated rats caused a significant increase (*p* < 0.0001) in the activities of CAT and SOD, besides GSH levels, when compared to the DMBA group. However, it still significantly decreased (*p* < 0.0001) compared to control rats. The same results were obtained for GST enzymatic activities (*p* < 0.001). Furthermore, a significant increase (*p* < 0.0001) in the MDA concentration was observed in DMBA-intoxicated rats compared to their counterparts in the control group. While MDA concentrations were significantly decreased in DMBA-intoxicated rats treated with GRV compared to the DMBA group, they still significantly increased (*p* < 0.0001) compared to the control group. In contrast, there was no discernible difference between the control group and the GRV-treated rats.

**TABLE 6:** The therapeutic impact of graviola (GRV) on lipid peroxidation represented as malondialdehyde (MDA) and antioxidant’s activities of CAT, SOD, GST, and GSH of rats intoxicated with DMBA and treated with GRV compared to control rats.

Group	CAT (U/mg protein)	SOD (U/mg protein)	GST (U/mg protein)	GSH (mmol/mg protein)	MDA (nmol/g tissue)
Control	39.58 ± 1.059^a^	28.47 ± 1.26^a^	9.80 ± 0.37^a^	23.43 ± 0.94^a^	19.59 ± 1.96^c^
GRV	39.83 ± 1.94^a^	28.79 ± 5.21^a^	9.63 ± 0.83^a^	22.93 ± 1.30^a^	19.44 ± 2.23^c^
DMBA	14.23 ± 1.03^c^	11.43 ± 1.33^c^	4.10 ± 0.67^c^	9.80 ± 0.85^c^	64.05 ± 0.72^a^
DMBA + GRV	28.72 ± 0.63^b^	19.61 ± .87^b^	6.78 ± 0.89^b^	16.96 ± 1.92^b^	42.24 ± 1.63^b^
*p*-value	0.0001	0.0001	0.001	0.0001	0.0001

Data presented as means ± SEM, for ten rats in each group. The significant change was assessed at *p* < 0.05. Means within the same column, which carry different superscript letters (a, b and c)**,** are significantly different (*p* < 0.01). (N = 10). SOD: superoxide dismutase; GST:Glutathione S Transferase; GSH: glutathione reduced; MDA: malondialdehyde. DMBA: 7, 12-Dimethylbenz (a) anthracene; GRV: graviola.

### 3.4 The therapeutic impact of GRV on CYP2E1 and pro-inflammatory cytokines (TNF-α and IL-β1) mRNA expression

**FIGURE 1:**
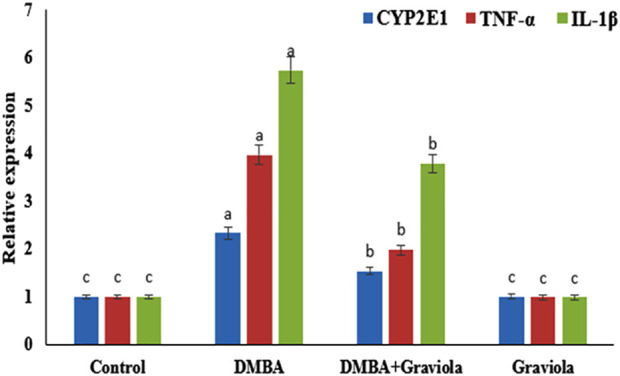
Relative gene expression of CYP2E1, TNF-α and IL-1β mRNA in renal tissue of control and treated groups. Means within the same figure, which carry different superscript letters, are significantly different (*p* < 0.01). CYP2E1: Cytochrome P450 Family 2 Subfamily E Member 1; TNF-α: tumour necrosis factor alpha; IL-1β:interleukin-1-beta.

Data shown in Figure 1 revealed a significant increase (*p* < 0.0001) in mRNA expression levels of CYP2E1, TNF-α, and IL-1β in the DMBA groups compared to the control rats. DMBA-intoxicated rats treated with oral administration of GRV showed a significant reduction (*p* < 0.0001) in the mRNA expression of CYP2E1, TNF-α, and IL-1β compared to the corresponding DMBA groups. However, they still significantly increased (*p* < 0.0001) in comparison to the control group.

### 3.5 Histopathological assessment

**FIGURE 2:**
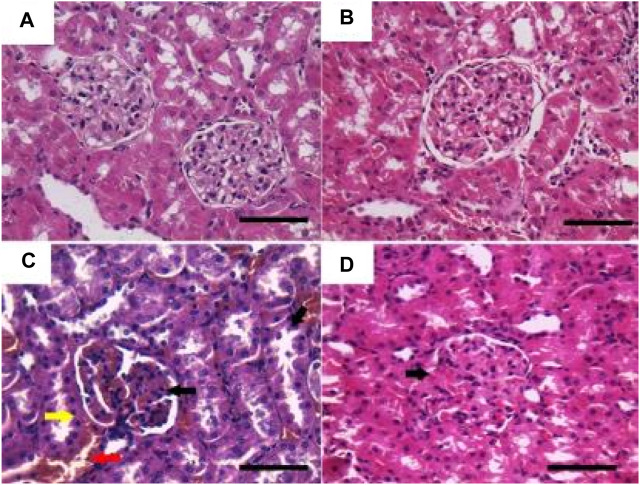
Representative photomicrographs showing the histopathological lesions of renal tissues of (a) control, (b) GRV-treated, (c) DMBA -treated, and (f) DMBA + GRV-treated rats stained with H&E; scale bar = 50 µm: **(A, B)** Normal glomeruli, renal tubules, renal epithelium, and interstitium. **(C)** Dilatation and congestion of intertubular (red arrow) and glomerular (black arrow) blood vasculature, attenuation and degeneration of renal epithelium, nuclear pyknosis, and cytoplasmic eosinophilia of renal epithelial cells (yellow arrow). **(D)** Marked improvement in the histological structure of renal tubules and renal epithelium with mild glomerular congestion (black arrow).

As shown in Figure 2, the histopathological examination of rats’ kidneys in the control and GRV groups revealed normal glomeruli, renal tubules, renal epithelium, and interstitium (Figure 2 a,b). While DMBA-intoxicated rats showed dilatation and congestion of intertubular and glomerular blood vasculature, attenuation and degeneration of renal epithelium, nuclear pyknosis, and cytoplasmic eosinophilia of several renal epithelial cells (Figure 2C), In addition, several sections showed extravasation of erythrocytes into the surrounding interstitium and intertubular lymphocytic infiltration. On the contrary, rats in the DMBA + GRV group showed marked improvement in the histological structure of renal tubules and renal epithelium with mild glomerular congestion (Figure 2D). Semiquantitative scoring for different pathologic lesions revealed a significant increase in the DMBA-intoxicated group compared to the control group. However, a significant decrease in the means of these scored lesions was reported in the DMBA + GRV group compared to the DMBA-intoxicated group, although it was not identical for the control group (Table 7).

**TABLE 7:** The semiquantitative lesion scores of rats’ kidney treated with graviola (GRV), DMBA: 7, 12-Dimethylbenz (a) anthracene, or its combination.

Scored lesions	Control	GRV	DMBA	DMBA + GRV
Vascular congestion and hemorrhage	+	+	+++	++
degeneration of renal epithelium	-	-	+++	+
necrosis of renal epithelium	-	-	++	+
intertubular lymphocytic infiltration	-	-	++	+

Four levels of severity were used in the scoring system: no histologic change (−), mild (+), moderate (+), and severe (+++) pathologic changes. The individual score for each rat was estimated, and then the grading decision was determined by calculating the median score for each group.

**FIGURE 3:**
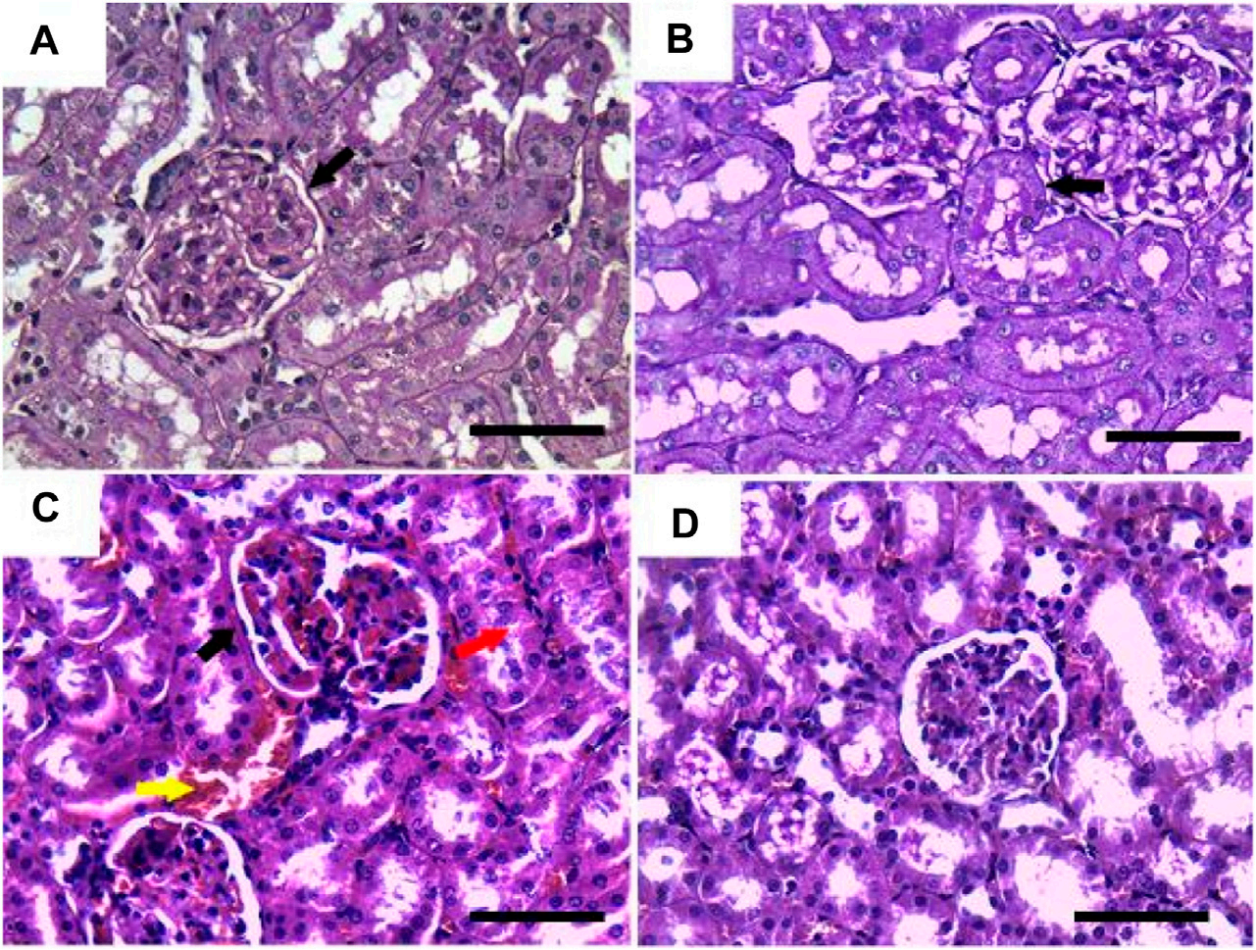
Representative photomicrographs showing the histopathological lesions of renal tissues of (a) control, (b) GRV-treated, (c) DMBA -treated, and (f) DMBA + GRV-treated rats stained with PAS; scale bar = 50 µm: **(A, B)** Positive reaction in the glomerular basement membrane and apical brush borders of renal tubules (black arrows). **(C)** Thickened and wrinkly glomerular and tubular basement membranes (black arrow), with partial distruction of brush borders (red arrow) and intertubular congestion (yellow arrow). **(D)** Nearly normal shape and thickening of the glomerular and tubular basement membranes, as well as tubular brush borders.

On the other hand, sections stained with PAS showed a positive reaction in the glomerular basement membrane and apical brush borders of renal tubules in the control and GRV groups (Figures 3A, B). However, PAS-stained sections from DMBA-treated rats showed positive stained, thickened, and wrinkly glomerular and tubular basement membranes, with partial destruction of brush borders (Figure 3C). A nearly normal shape and thickening of the glomerular and tubular basement membranes and tubular brush borders were observed in DMBA + GRV rats (Figure 3D). The quantitative scoring for PAS staining density revealed a significant increase in the DMBA-intoxicated group due to the thickening of basement membranes compared to control rats. However, a significant decrease in the optical density of PAS-positive tissues was reported in the DMBA + GRV group compared to the DMBA-intoxicated group, albeit it was not identical for the control group (Figure 8A).

**FIGURE 4:**
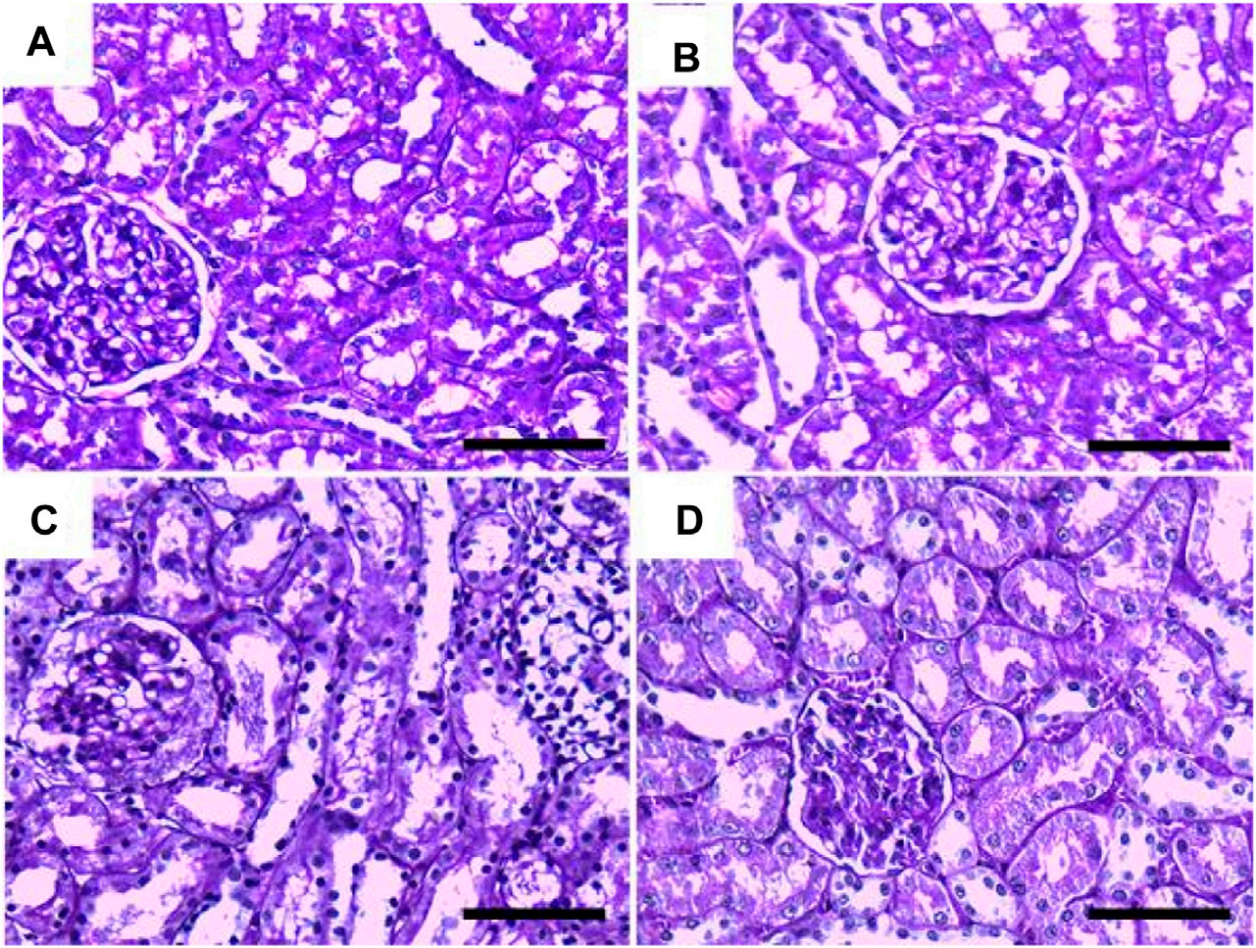
Representative photomicrographs showing the histopathological lesions of renal tissues of (a) control, (b) GRV-treated, (c) DMBA -treated, and (f) DMBA + GRV-treated rats stained with alcian blue/PAS; scale bar = 50 µm: **(A, B, C, D)** Positive reactions in renal glomeruli and renal tubules without marked or noticeable changes between tissues from different groups.

In Figure 4, Albican Blue/PAS staining for control, intoxicated, and treated rats showed positive reactions in the renal glomeruli and renal tubules without marked or noticeable changes in distribution and expression pattern of mucins between tissues from different groups. The quantitative scoring for Albican Blue/PAS staining density revealed non-significant changes between control and treated rats in different groups (Figure 8B).

### 3.6 Immunohistochemical findings

**FIGURE 5:**
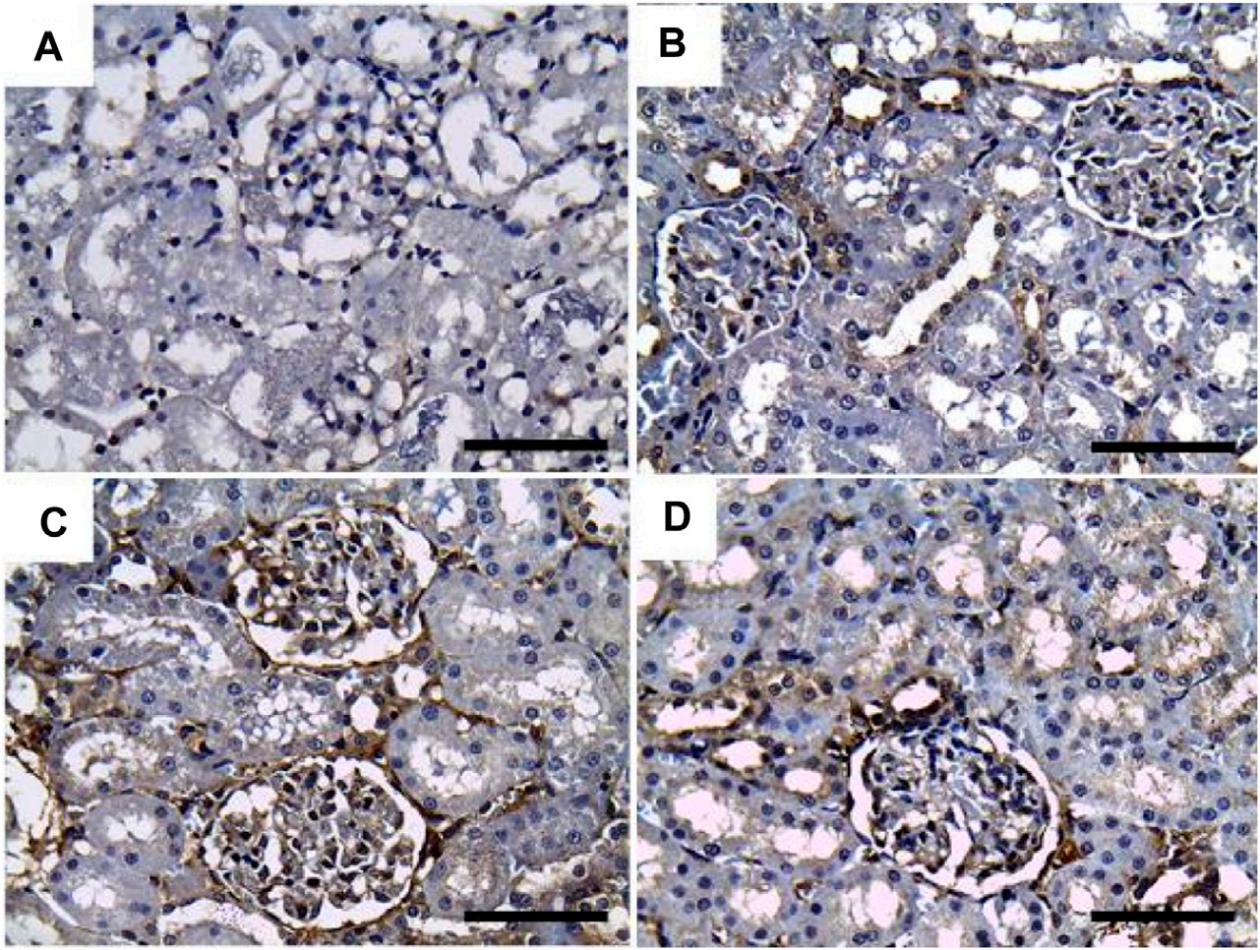
Representative photomicrographs showing the immunohistochemical expression of TNF-α in renal tissues of (a) control, (b) GRV-treated, (c) DMBA -treated, and (f) DMBA + GRV-treated rats; scale bar = 50 µm: **(A)** Negative immunostaining against TNF-α. **(B)** Mild glomerular and tubular immunoreactivity. **(C)** Moderate to intense glomerular and tubular immune reactions. **(D)** Decreased glomerular and tubular immune reactivity compared to their counterparts in the DMBA-treated group.

As illustrated in Figure 5, negative immunostaining against TNF-α was reported in the control group (Figure 5A). However, the immunohistochemical examination of the kidneys of rats treated with GRV showed mild glomerular and tubular immunoreactivity (Figure 5B). On the contrary, moderate to intense glomerular and tubular immune reactions were noticed in the DMBA-treated group (Figure 5C). Rats in the DMBA + GRV-treated group revealed decreased glomerular and tubular immune reactivity compared to their counterparts in the DMBA-treated group (Figure 5C). The quantitative scoring for TNF-α- immunoreacted cells revealed a significant increase in kidneys in the DMBA-intoxicated group compared to control rats. However, a significant decrease in the optical density of TNF-α immunopositive cells was reported in the DMBA + GRV group compared to the DMBA-intoxicated group, although it was not identical for the control group (Figure 8C).

**FIGURE 6:**
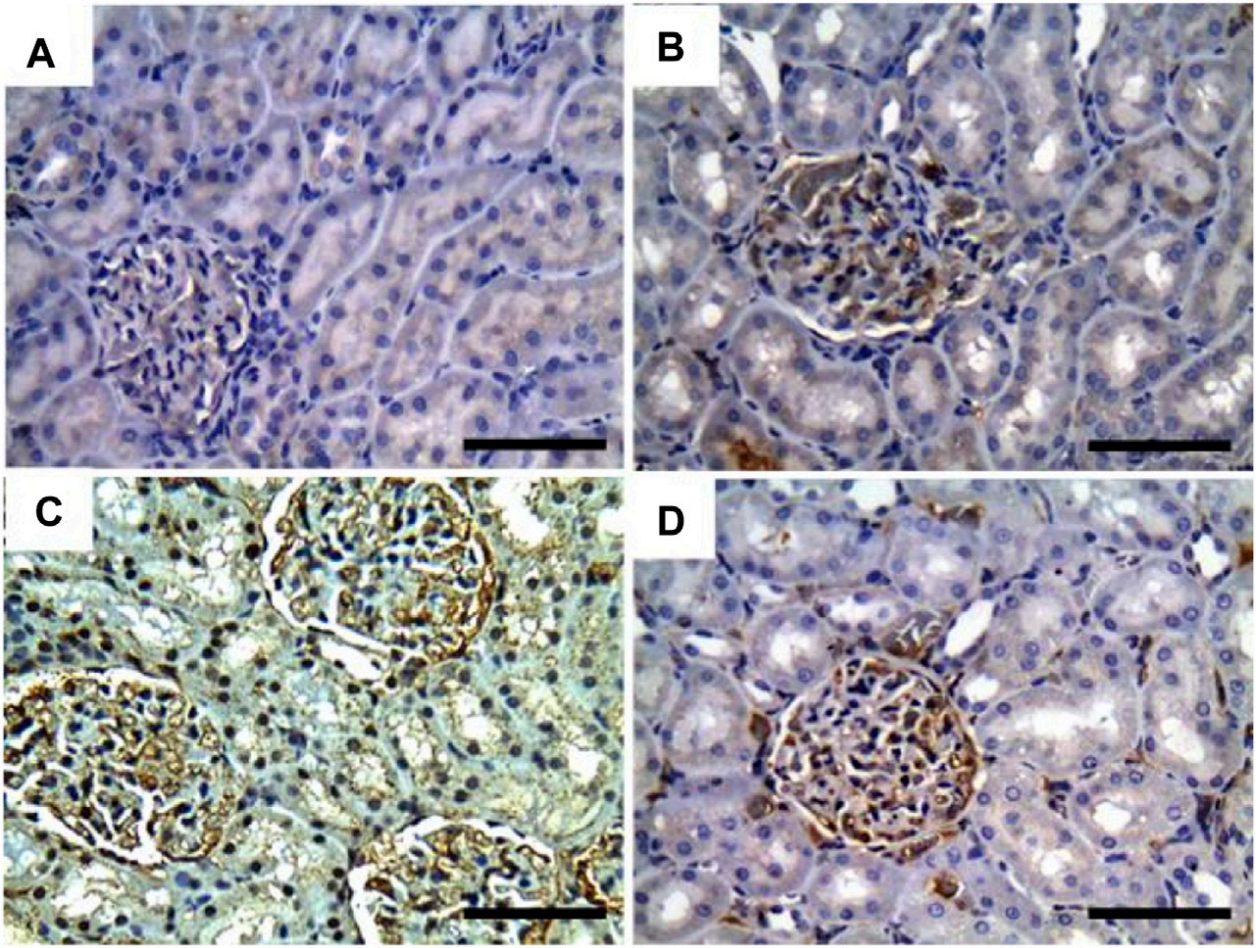
Representative photomicrographs showing the immunohistochemical expression of IL1-β in renal tissues of (a) control, (b) GRV-treated, (c) DMBA -treated, and (f) DMBA + GRV-treated rats; scale bar = 50 µm: **(A)** Negative glomerular and tubular immunoreactivity. **(B)** Mild glomerular immunoreactivity against IL-1β. **(C)** Intense immunostaining of the glomerulus and renal tubules. **(D)** Moderate glomerular immunoreactivity.

Additionally, Figure 6 showed that the immunoexpression of IL-1β in the control group revealed negative glomerular and tubular immunoreactivity (Figure 6A). However, mild glomerular immunoreactivity against IL-1β was reported in the GRV-treated group (Figure 6B). On the contrary, intense immunostaining of the glomerulus and renal tubules was noticed in DMBA-intoxicated rats (Figure 6C). While rats in DMBA + GRV-treated rats showed moderate glomerular immunoreactivity (Figure 6D), The quantitative scoring for IL-1β-immune-reacted cells revealed a significant increase in kidneys in the DMBA-intoxicated group compared to control rats. However, a significant decrease in the optical density of IL-1β-immunopositive cells was reported in the DMBA + GRV group compared to the DMBA-intoxicated group, albeit it was not identical for the control group (Figure 8D).

**FIGURE 7:**
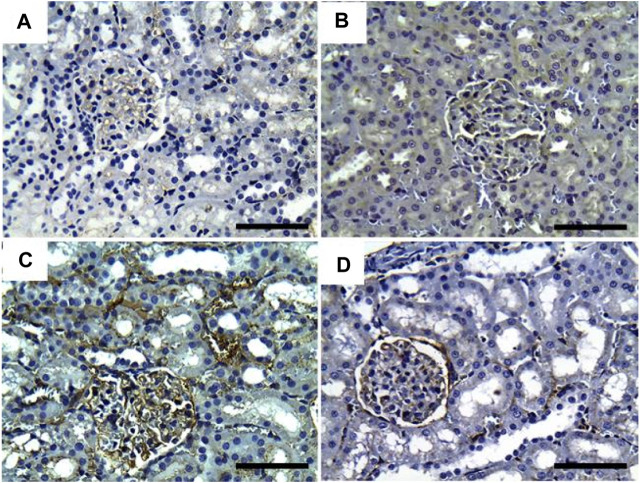
Representative photomicrographs showing the immunohistochemical expression of CYP450 in renal tissues of (a) control, (b) GRV-treated, (c) DMBA-treated, and (f) DMBA + GRV-treated rats; scale bar = 50 µm: **(A, B)** Negative immunoreactivity against CYP2E1. **(C)** Moderate to intense glomerular and tubular immunoexpression. **(D)** Mild to moderate glomerular immunoreactivity against CYP450.

**FIGURE 8:**
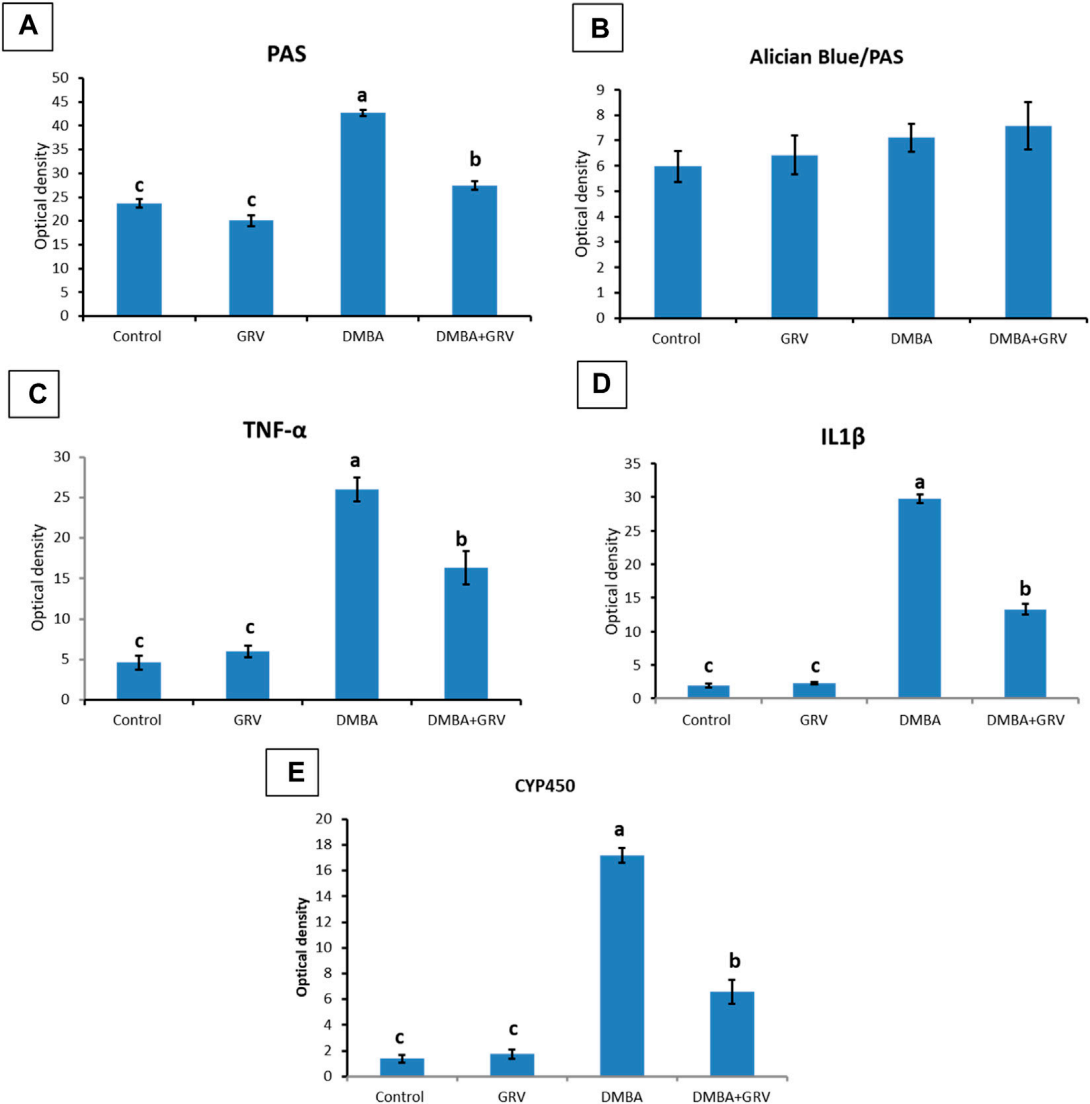
Quantitative histomorphometric analysis for staining optical density in renal tissues stained with: **(A)** PAS, **(B)** Alician Blue/PAS, **(C)** TNF- α, **(D)** IL-1β, and **(E)** CYP450. Means within the same figure, which carry different superscript letters, are significantly different (*p* < 0.01). CYP 450: Cytochrome P450 F; TNF-α: tumour necrosis factor alpha; IL-1β:interleukin-1-beta.

Regarding CYP2E1 immune expression, control and GRV rats showed negative immunoreactivity against CYP2E1 (Figures 7A, B). On the contrary, moderate to intense glomerular and tubular immunoexpression was noted in DMBA-intoxicated rats (Figure 7C). However, mild to moderate glomerular immunoreactivity against CYP2E1 was observed in DMBA + GRV-treated rats (Figure 7D). The quantitative scoring for CYP2E1-immune-expressed cells revealed a significant increase in kidneys in the DMBA-intoxicated group compared to control rats. However, a significant decrease in the optical density of CYP2E1-immunopositive cells was reported in the DMBA + GRV group compared to the DMBA-intoxicated group, although it was not identical for the control group (Figure 8E).

## 4 Discussion

*Annona muricata L.* (soursop or graviola) is a widely known tropical plant consumed by the community. It is also traditionally used to treat diverse ailments such as fever, pain, respiratory illness, infection, diabetes, cancer, and hypertension (Mutakin et al., 2022). The family Annonaceae has a long history of being recognized and used as a natural herbal treatment all throughout the world (Moghadamtousi et al., 2015a). Consuming graviola significantly improves human health (Saleem et al., 2017). It is therapeutically used for anticancer, antibacterial, antiviral, anti-fungal, anti-malarial, anti-tumor, anxiolytic, anti-stress, analgesic, hypoglycemic, hypotensive, hepatoprotective, gastroprotective, anti-inflammatory, and immunomodulatory properties (Chaparro et al., 2014; Gavamukulya et al., 2017). However, few reports are available concerning their beneficial impacts against renal injury. Polycyclic aromatic hydrocarbons (PAHs), one type of atmospheric pollutant, are widely dispersed in the environment and enter human bodies through food, drink, and the air. DMBA, an PAH environmental pollutant, has several harmful and carcinogenic consequences (Kleiner et al., 2002). Previously, DMBA treatment increased urea and creatinine serum concentrations due to an upregulation of protein catabolism (Burchiel et al., 1990). Meanwhile, urea represents the nitrogenous waste produced by protein degradation (Akhouri et al., 2020). As a result, elevated urea and creatinine levels in the serum indicate renal disease (Krishnamoorthy and Sankaran, 2016). While the increased serum uric acid level indicates nephrotoxicity (Burchiel et al., 1990), which can indicate kidney impairment (Akhouri et al., 2020). The current study reported a significant increase in the concentration of urea, creatinine, and uric acid in the serum of the DMBA-treated group. On the other side, the DMBA + GRV-treated group showed enhanced renal functions. The potential efficacy of graviola is due to extensive phytochemical evaluations on different parts of the *A. muricata* plant, which have shown the presence of various phytoconstituents and compounds, including alkaloids (Yang et al., 2015), megastigmanes (Nawwar et al., 2012), flavonol triglycerides (Nawwar et al., 2012), phenolics (Jimenez et al., 2014), cyclopeptides, and essential oils (Pélissier et al., 1994; Kossouoh et al., 2007). However, Annona species, including *A. muricata*, are a generally rich source of annonaceous acetogenin compounds (Rupprecht et al., 1990) and major minerals such as K, Ca, Na, Cu, Fe, and Mg, suggesting that regular consumption of *A. muricata* fruit can help provide essential nutrients and elements to the human body (Gyamfi et al., 2011).

Oxidative stress is the main cause of nephrotoxicity (Kasprzak, 2023), where the reactive oxygen metabolites are formed by the mitochondria of the renal cortex (Padmini and Kumar, 2012). However, the toxicity of the glomerular and tubular epithelial cells may be induced by mechanisms compromising the mitochondria’s regular cellular functions and/or the integrity of their membranes (Dakrory et al., 2015b), which induces renal injury (Du and Yang, 1994). Early research suggests that DMBA stimulates the generation of reactive oxygen species (ROS), which damages DNA, causes lipid peroxidation, and depletes the antioxidant defense mechanisms in cells (Dakrory et al., 2015b). ROS affect mesangial and endothelial cells, which can result in oxidative stress and alter the glomerulus' appearance and function (Kandeel et al., 2011; Sharma et al., 2012). When the amount of reactive oxygen species (O_2_, H_2_O_2_, and -OH) generated exceeds the antioxidant capacity of the cells, oxidative damage results (Kesba and El-Beltagi, 2012). The damage caused by the ROS generated during DMBA metabolism may spread from the site of formation to other locations inside or even outside the cells (Koul et al., 2010). Reactive carbonyl compounds, the most common form of malondialdehyde, are a complicated series of molecules that can be formed when lipid peroxides produced from the disintegration of polyunsaturated fatty acids (Akande and Akinyinka, 2005; Gandhi et al., 2017). According to the current study, MDA levels significantly increased in the DMBA-treated group compared to the control groups; these results are consistent with Sharma and Paliwal (2014). However, the MDA concentration significantly dropped in the GRV-treated group. This decrease demonstrated the extract’s capacity to counteract the oxidative damage caused by DMBA. Graviola’s antioxidant capability clarified the normalization effect in the DMBA + GRV treatment group. Few studies have described the polyphenols found in *A. muricata* leaves. The leaves of A. muricata were shown to contain kaempferol, quercetin-glucoside, quercetin, and rutin in studies by Yang et al. (2015) and Oliveira et al. (2017). Nevertheless, the majority of research examined crude extracts; the greater antioxidant potential of aquas and methanolic extracts of A. muricata may be explained by the high concentration of phenolic components in these extracts. Numerous studies have demonstrated that the chemicals discovered by HPLC analysis, including rutin, quercetin, procyanidins B2 and C1, (epi) catechin, and kaempferol, are strong antioxidants. These compounds possess the ability to produce stable antioxidant-derived radicals, as well as the ability to scavenge free radicals and reduce hydrogen or donate electrons (Cos et al., 2003; Gu et al., 2006; Vellosa et al., 2011; Ganeshpurkar and Saluja, 2017). The antioxidant potential of *A. muricata* leaves has also been documented in earlier *in vitro* and *in vivo* investigations (Florence et al., 2014; Moghadamtousi et al., 2015b; Olas, 2023), which supports our findings. Moreover, Baskar et al. (2007) reported that GRV has a defensive ability against free radicals (OH) and H_2_O_2_. Consequently, it blocks the elevation of LPO (Spitz et al., 2004) and converts the ROS into harmless properties (Ekaluo et al., 2016).

Enzymatic and non-enzymatic antioxidants are endogenous antioxidants that can combat ROS and lessen oxidative stress. A number of metabolic pathways changed, along with alterations in lipid peroxidation-generating processes and antioxidant defense mechanisms (Paliwal et al., 2011). Catalase, glutathione-S-transferase, superoxide`e dismutase (SOD), and other enzymes have been suggested as indicators for oxidative stress (Bedi and Priyanka, 2012; Sullivan et al., 2020). In the current study, the oxidative stress induced by DMBA is indicated by a significant reduction in CAT, SOD, and GST, where these enzymes were blocked by the excessive ROS production. This adverse oxidative impact is countered by the increased levels of antioxidant enzymes, which were related to GRV treatment. These enzymes stop the production of hydroxyl radicals and guard the components of the cell against oxidative damage (Gill and Tuteja, 2010; Wu et al., 2011). Flavonoids and other phenolic acids included in GRV are known to inhibit the oxidation of cellular macromolecules via their radical scavenging properties or by activating ROS-reducing efficacy, which is attributed to the upregulation of the antioxidant genes (Jiménez-Osorio et al., 2015). Moreover, Baskar et al. (2007) concluded that GRV possesses potent antioxidant activities due to the presence of acetogenins, which can play a crucial role in ROS scavenging. These findings are consistent with Sharma and Paliwal (2013) and Mohamed et al. (2021).

In previous research, it has been proposed that the progression of renal injury depends on pro-inflammatory cytokines produced in renal tissues (Navarro and Mora-Fernández, 2006). In the present study, DMBA increased the gene expression of TNF-α, IL1β, and CYP2E1 as compared to the control group. The enhancement of cytokines secretion from various cells enhances the expression of COX2, which is known as an enzyme to produce a variety of inflammatory mediators like leukotrienes (LTs), prostaglandins, and others (Sarbishegi et al., 2016; Hong et al., 2021). Recently, it was concluded that treatment with GRV extract reduced inflammatory cytokines (IL-6, IL8) (Hong et al., 2021) and inhibited both COX-1 and COX-2 (Rady et al., 2018). These findings suggested the crucial role of GRV in the alleviation of inflammatory responses in renal tissues via inhibiting COX-2 as well as proinflammatory cytokines (Helal and Abd Elhameed, 2021). Data from the current study support these findings, where rats from the DMBA + GRV group showed a significant decrease in levels of TNF-α, IL1β, and CYP2E1 compared to the group that received DMBA alone. The presence of anti-inflammatory substances (alkaloids, saponins, flavonoids, and tannins) in graviola extracts may prevent the formation of prostaglandins, which retain the expression levels of TNF-α, CYP2E1, and IL1β to the normal level (Serafini et al., 2010). Several studies are still needed to explore the anti-inflammatory power of the main individual constituents of GRV.

DMBA-induced oxidative stress induces damage to lipids, proteins, and nucleic acids, leading to interconnected disruptions of cellular metabolism (Krishnamoorthy and Sankaran, 2016). Therefore, proteinuria, renal tubular necrosis, and increased specific signals such as TNF-α, chemokines, and cytokines are all signs of DMBA-induced nephrotoxicity (Sharma and Paliwal, 2012). The histopathological and immunohistochemical examinations of the kidneys of the different experimental groups supported the results of antioxidant status and oxidative stress markers. DMBA produced marked histopathological alterations in the kidney, including dilation of tubules and sloughing of the epithelium, which indicates advanced disintegration of tubules (Sharma et al., 2012). Treatment with GRV leaf extract normalizes nephrotoxicity’s histolopathological abnormalities (Sabra and Ahmed, 2018). In contrast, GRV administered to DMBA-induced rats protected renal tissue from degenerative changes that are mediated by oxidative stress. This protective effect of GRV leaves may be due to its anti-inflammatory action and its ability to neutralize free radicals due to the presence of flavonoids and other polyphenols (Sabra and Ahmed, 2018).

Results from PAS and Ablican Blue/PAS-stained sections revealed interstitial lymphocytic infiltration, hemorrhage, thickened and wrinkled tubular basement membranes, and partial disruption of the brush border in DMBA-intoxicated rats. However, the DMBA + GRV-treated group showed marked attenuation of DMBA-related adverse impacts, in addition to positive reactions in the glomerular basement membrane and apical microvilli of renal tubules. Similar results were previously reported by Yen-Nien et al. (2019). In addition, the immunohistochemical examination of TNF-α, CYP2E1, and IL-β1 in DMBA-intoxicated groups showed a significant increase in the immunohistochemical expression of the examined inflammatory cytokines. However, concurrent treatment with GRV revealed a reduction of these inflammatory cytokines, which revealed its efficient anti-inflammatory effect. These findings was supported by Mitsui et al. (2015), which demonstrated that cytochrome P450 1B1 (CYP1B1) has been upregulated in many types of cancer, including renal cell carcinoma. Also, Sonoda et al. (2015) studied the levels of TNF receptors 1 and 2 (TNFR1 and TNFR2) in serum and urine, which were associated with other markers of kidney injury and renal histological findings. Further studies are still needed to explain the anti-inflammatory impact of GRV and its related mechanisms.

## 5 Conclusion

Based on the findings mentioned above, it could be concluded that GRV is a naturally beneficial medicinal product that can alleviate the renal toxicity resulting from environmental exposure to DMBA. The reno-protective effects offered by the GRV may involve its anti-inflammatory, antioxidant, and/or oxidative free radical scavenging properties. The antioxidant activities of GRV are based on the presence of phytochemical compounds such as kaempferol, quercetin-glucoside, quercetin, and rutin, which were proved to be strong antioxidants due to their abilities to scavenge ROS, reduce hydrogen or donate electrons, and produce stable antioxidant-derived radicals. Additionally, the crucial anti-inflammatory role of GRV was proved by their abilities to inhibit COX-2 and proinflammatory cytokines due to the presence of anti-inflammatory substances (alkaloids, saponins, flavonoids, and tannins) in GRV extracts, which prevent the formation of prostaglandins and retain the expression levels of TNF-α, CYP2E1, and IL1β to the normal levels. Therefore, the useful effect of GRV appeared when consumed for long periods and was preferred as a protective agent against polycyclic aromatic hydrocarbons, particularly 7,12-dimethylbenz[a]anthracene (DMBA).

## Data Availability

The original contributions presented in the study are included in the article/Supplementary Material, further inquiries can be directed to the corresponding author.
